# Losing One's Hand: Visual-Proprioceptive Conflict Affects Touch Perception

**DOI:** 10.1371/journal.pone.0006920

**Published:** 2009-09-07

**Authors:** Alessia Folegatti, Frédérique de Vignemont, Francesco Pavani, Yves Rossetti, Alessandro Farnè

**Affiliations:** 1 UMR-S 864 “Espace et Action,” INSERM, Bron, France; 2 Department of Psychology, University of Turin, Turin, Italy; 3 Hospices Civils de Lyon, Hôpital Neurologique, Mouvement et Handicap, Lyon, France; 4 Institut Jean Nicod, EHESS-ENS-CNRS, Paris, France; 5 Transitions, NYU-CNRS, New York, New York, United States of America; 6 Department of Cognitive Sciences and Education, University of Trento, Trento, Italy; 7 Center for Mind/Brain Sciences (CIMeC), University of Trento, Trento, Italy; 8 Université Claude Bernard Lyon I, Lyon, France; University of Sydney, Australia

## Abstract

**Background:**

While the sense of bodily ownership has now been widely investigated through the rubber hand illusion (RHI), very little is known about the sense of disownership. It has been hypothesized that the RHI also affects the ownership feelings towards the participant's own hand, as if the rubber hand replaced the participant's actual hand. Somatosensory changes observed in the participants' hand while experiencing the RHI have been taken as evidence for disownership of their real hand. Here we propose a theoretical framework to disambiguate whether such somatosensory changes are to be ascribed to the disownership of the real hand or rather to the anomalous visuo-proprioceptive conflict experienced by the participant during the RHI.

**Methodology/Principal Findings:**

In experiment 1, reaction times (RTs) to tactile stimuli delivered to the participants' hand slowed down following the establishment of the RHI. In experiment 2, the misalignment of visual and proprioceptive inputs was obtained via prismatic displacement, a situation in which ownership of the seen hand was doubtless. This condition slowed down the participants' tactile RTs. Thus, similar effects on touch perception emerged following RHI and prismatic displacement. Both manipulations also induced a proprioceptive drift, toward the fake hand in the first experiment and toward the visual position of the participants' hand in the second experiment.

**Conclusions/Significance:**

These findings reveal that somatosensory alterations in the experimental hand resulting from the RHI result from cross-modal mismatch between the seen and felt position of the hand. As such, they are not necessarily a signature of disownership.

## Introduction

In everyday life, there is little doubt that the body that I feel is my own. I know where my body parts lie in space and I know that every tactile sensation I feel comes from my body surface, irrespective of whether I see it or not. In the last decade the phenomenon of the rubber hand illusion (RHI) has called these certainties into question [1]. When their hand is hidden from view and is stroked in synchrony with a visible rubber hand, participants typically feel the strokes as if they originated from the location where the rubber hand is stroked. Furthermore, they can experience an illusory feeling of ownership towards the rubber hand. By contrast, asynchronous stroking of the rubber hand and the real hand prevents or abolishes the illusion (e.g. [2]; for a review see [3]). The displacement of touch onto the rubber hand is thought to be determined by a mechanism of visual capture of the tactile percept [4], [5]. Synchronous brushing induces a conflict between visual and proprioceptive information, which is partially solved in favor of vision [6], provided that prior knowledge about the body is not violated (e.g.[5], [2], [7]). Accordingly, the RHI is typically followed by a proprioceptive drift of the felt own hand position towards the rubber hand (e.g.[8]).

While the sense of bodily ownership has been now widely investigated through an impressive series of RHI studies, little is still known about the sense of disownership. There is indeed no experimental set-up that can artificially induce the explicit sensation of disownership of one's own hand. Body part disownership has mainly been described in the neuropsychological literature, especially after lesions of the right hemisphere [9], [10]. Disownership of contralesional limbs has often been associated with neglect, hemiplegia and somatosensory deficits, although these three disturbances are dissociable. Moro and colleagues [11], for example, described two patients suffering both from tactile extinction and somatoparaphrenia. The patients denied ownership of their contralesional limbs and reported that their limbs belonged to someone else (for review see [12]). Changing hand position in space improved left-sided tactile extinction without reducing the feeling of disownership, thus providing evidence that the neural substrates underlying the two symptoms are at least partly separated. Another case study that highlights the complexity of the relationship between disownership and tactile perception (for discussion see [13]) has been reported by Bottini and colleagues [14] (see also [15]). They described a patient suffering from denial of ownership of the left arm, who consistently attributed the left arm to her niece. Although she was unable to perceive touches on her left arm in normal conditions, she was able to detect tactile stimulations when the experimenter pretended to touch “her niece's hand”.

In order to provide better understanding of disownership phenomena in brain damaged patients, it would be of great interest to be able to induce and manipulate disownership in healthy participants. Interestingly, it has been recently suggested that the RHI is not only an ownership illusion, but also a disownership illusion [16], [17]. In particular, researchers have started to ask whether the sense of ownership felt towards the rubber hand can be accompanied by a sense of disownership towards the real hand. Put another way, is the rubber hand merely perceived as a third supernumerary limb, or does it somehow replace the participant's real hand? Longo and colleagues [16] ran a principal-components analysis on a detailed RHI questionnaire and revealed a component which comprised items reflecting paralysis of the hand and its disappearance. They assumed that this component indicates changes in the participants' feelings about their own hand and lack of agency over it, and accordingly called it ‘*loss of own hand*’. However, they underlined that this component only explained a small proportion of the variance of the RHI phenomenon. In addition, agreements with the statements concerning the loss of one's own hand were rather weak. Along the same line, Moseley and colleagues [17] showed a decrease in skin temperature of the real hand following the RHI. In addition, they showed that after having the right hand and the rubber hand stroked, participants required a larger inter stimulus interval to correctly determine the order of two tactile stimuli delivered to the left and right hands in a Temporal Order Judgment (TOJ) task, as if the denial of one's own hand decreased the weighting of tactile information felt on this hand. The authors interpreted the physiological variation and the somatosensory change in terms of functional disownership, suggesting that the participant's real hand was replaced by the artificial counterpart. As no detailed questionnaire was reported in the study, it remains to be established whether implicit somatosensory changes are accompanied by explicit feelings of disownership.

**Figure 1:**
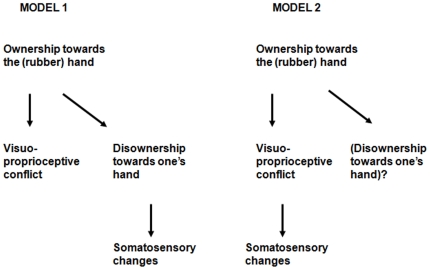
Alternative models for somatosensory changes.

The consequences and significance of the RHI for the participant's real hand are thus only partially understood. Somatosensory changes in the real hand might result from disownership, if there is disownership .Alternatively, rather than disownership they might reflect the anomalous visuo-proprioceptive conflict experienced by the participant during the RHI. In the present study, we examined whether changes in tactile perception should be attributed to disownership or to the visuo-proprioceptive conflict. In the former case, any effect on somatosensation should be specific to manipulations that alter body part ownership, such as the RHI. In the latter case, effects on somatosensation should also be found in other situations of visuo-proprioceptive conflict in which body part ownership is not altered (see Figure 1). Such situation can be obtained when the real hand is perceived through prismatic lenses, which introduce a conflict between hand position experienced through proprioception and hand position as indicated by vision, without introducing any uncertainty about the body part ownership. To test our hypotheses, we used a speeded simple detection task of suprathreshold electro-cutaneous stimuli to assess the ability to report tactile stimulation on the real hand after induction of the RHI (Experiment 1) and while wearing prismatic goggles (Experiment 2).

## Materials and Methods

### Subjects

Fifteen (10 females, mean age 25.8 years; 12 right-handed and 3 left-handed by self-report) and ten (6 females, mean age 25.6 years, right handed) healthy subjects participated in Experiment 1 and 2, respectively. All gave their verbal informed consent to participate in the study, which was approved by the INSERM U864 ethics committee. All participants had normal or corrected-to-normal vision and normal touch perception by self report.

### Apparatus and procedures Experiment 1

**Figure 2:**
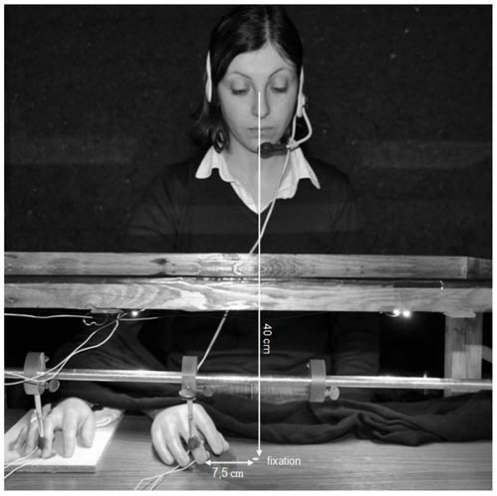
Set-up experiment 1. The picture represents an actor who consented to be photographed for illustrative graphical purposes. Viewing distance from the fixation point is reported, as well as the distance between the rubber hand middle finger and the fixation point (in cm).

Participants sat at a table in a sound-attenuated and dimly illuminated room, facing the experimental apparatus (see Figure 2). The participant's right hand was positioned on the table, beneath a wooden frame covered by a semi-silvered mirror. The rubber hand (a real-size prosthetic model of a right hand) was placed 7.5 cm to the right of the body midline and 15 cm to the left of the concealed right hand (all measures taken with respect to the stimulated middle finger). The rubber hand was slightly oriented anti-clockwise, to maximize posture plausibility with respect to the participant's right elbow. A fixation point was marked on the table along the body midline, 7.5 cm to the left of the rubber hand. The rubber hand was thus visible in peripheral vision, unlike most previous studies (but see [5]). At a viewing distance of about 40 cm, the distance between the stimulated finger on the rubber hand and the fixation point subtended an angle of about 10.5 degrees.

### Tactile task

Supra-threshold electro-cutaneous stimuli (DS7A, Digitimer) were delivered to the participant's right middle fingertip via disposable neurology electrodes (Neuroline). Stimulation was controlled by a computerized script (xgen; www.sph.sc.edu/comd/rorden). Prior to the experimental session, a variable number of blocks of tactile stimuli (10 stimuli per block) was delivered to each participant for titration purposes (to obtain ≥80% of accurate detection)

The experimental block of tactile stimulation comprised 20 trials and lasted about 80 seconds. Each trial began with a warning sound (beep) after which a tactile stimulus or no stimulus (catch trials) followed randomly interleaved. Participants were instructed to maintain central fixation (monitored via an infrared webcam) and to respond vocally (“tah”) as soon as they felt a tactile stimulus. Their vocal reaction times (RT) were recorded by means of a voice key. During the tactile task, the lights under the mirror were switched on. This had the effect of making the rubber hand visible, whereas the participant's hand remained concealed from view.

### Proprioceptive judgment task

Immediately following the tactile task, participants were required to estimate the position of their hidden right middle-finger by means of a ruler reflected on the mirror and appearing to be at the same depth as the hands (see [2] for this method). They were instructed to report the number on the ruler corresponding to the position of their finger by mentally projecting a parasagittal line from the finger to the ruler. During proprioceptive judgments the lights under the mirror were switched off to make the rubber hand invisible while only the ruler was visible. Participants were required to repeat the judgment 6 times, with the ruler always presented with a random offset in order to avoid response strategies. The mismatch between the true position of the finger and the number indicated by the participant was calculated and resulted in a positive number if the displacement was towards the rubber hand and a negative number if it was away from it. This procedure allowed measuring the drift of the perceived position of the participant's own hand towards the rubber hand, a well established measure of the RHI [1], [2].

### Visuo-Tactile training

After the judgment the ruler was removed and the lights under the mirror were turned on to make the rubber hand appear. A two-minute stimulation with identical paintbrushes followed. Both the fake and real hands were manually stimulated with touches delivered along the dorsum of the middle-finger. Across separate conditions, brushes on the fake and real hand were delivered both synchronously and asynchronously, with a resting period of 5 minutes between the two conditions. Order of these two conditions was counterbalanced between participants.

Both the tactile and the proprioceptive judgment tasks were repeated after synchronous and asynchronous training. In sum, all participants underwent two experimental sessions, each composed of tactile task, proprioceptive judgment, visuo-tactile training, then repetition of tactile task and proprioceptive judgment in the same order.

### Questionnaire

At the end of each session, participants were also required to complete a questionnaire, rating their agreement with 12 statements describing the RHI experience. Six statements referred to the experience of embodiment of the rubber hand and addressed both the displacement of the tactile percept onto the fake hand and its ownership (e.g., “It seemed as if I were feeling the touch of the paintbrush in the location where I saw the rubber hand touched” or “I felt as if the rubber hand were my own hand”). The remaining six statements referred to the feeling of disownership of their own hand (e.g., “It seemed as if my right hand had disappeared”). Participants were asked to judge their level of agreement with each statement by drawing a mark on a 14 cm long continuous line in which the left extreme indicated complete disagreement and the right extreme indicated complete agreement. See questionnaire in Appendix S1.

### Apparatus and Procedures Experiment 2

The same experimental set-up and procedures of Experiment 1 were used in Experiment 2, unless otherwise stated. As in Experiment 1, participants underwent the tactile titration procedure and were then submitted to the same tactile task that was, however, performed in two different visual conditions: One in which they were wearing goggles fitted with neutral lenses, and one in which the goggles were fitted with prismatic lenses displacing the visual field leftwards by 15 degrees of visual angle. To control for any effect due to fatigue or habituation to the prisms, the tactile task was administered twice with neutral goggles, immediately before and after the tactile task performed while wearing the prismatic lenses. During these blocks, the light under the mirror were turned on and the participant saw his/her own hand (displaced or not, depending on the visual condition). Before and after each block of tactile task, the lights were switched off and participants were asked to perform 6 proprioceptive judgments of the position of their right middle finger. In addition, to check for any passive adaptation possibly induced by wearing the prisms, participants also made a series of 6 open-loop (no visual feedback from the moving hand) pointing movements to a visual target briefly presented in front of them, at the beginning and at the end of the blocks. No after-effects were expected, as no visuo-motor adaptation was induced.

**Figure 3:**
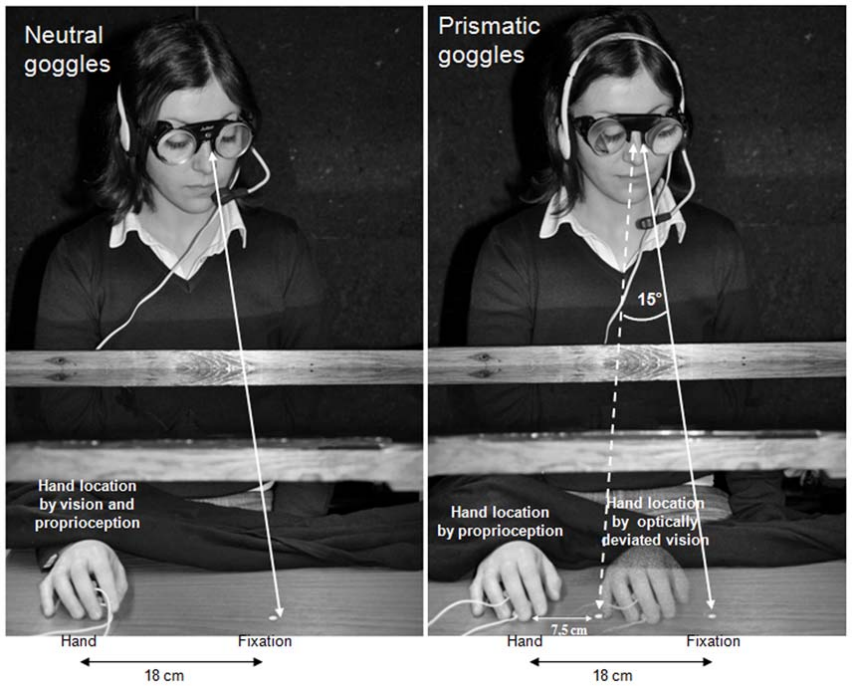
Set-up experiment 2. The pictures represent an actor who consented to be photographed for illustrative graphical purposes. The distance from the actor's real hand and the fixation point was 18 cm both in the neutral (left panel) and optically displaced condition (right panel). The shaded hand in the left panel represent the virtual location of the hand and fixation point, as seen by the subject trough prismatic goggles inducing a shift of about 15 degrees of visual angle.

It should be noted that the point of fixation differed between neutral and prismatic blocks, to make the distance between eye fixation and proprioceptive inputs comparable across visual conditions. This is illustrated in Figure 3. In the prismatic lenses condition participants saw their hand displaced about 10,5 cm to the left of its real position (due to the 15° prismatic displacement, at a viewing distance of about 40 cm). To ensure that in both conditions the eyes were fixating at the same distance relative to the proprioceptively specified position of the hand, the fixation point lay 18 cm left from the middle finger in the neutral lenses condition, and 7.5 cm (as in Experiment 1) from the middle finger in the prismatic lenses condition (i.e., 10.5 cm rightwards). This procedure was adopted because slower processing of tactile stimulation has been previously documented when the participant's attention or gaze has been diverted from the stimulated hand, even when hand vision is prevented [18]–[22]. While the questionnaire was not administrated in experiment 2, none of the participants spontaneously reported feeling as if her hand no longer belonged or “belonged less” to her.

## Results

### Experiment 1

#### Questionnaire

**Figure 4:**
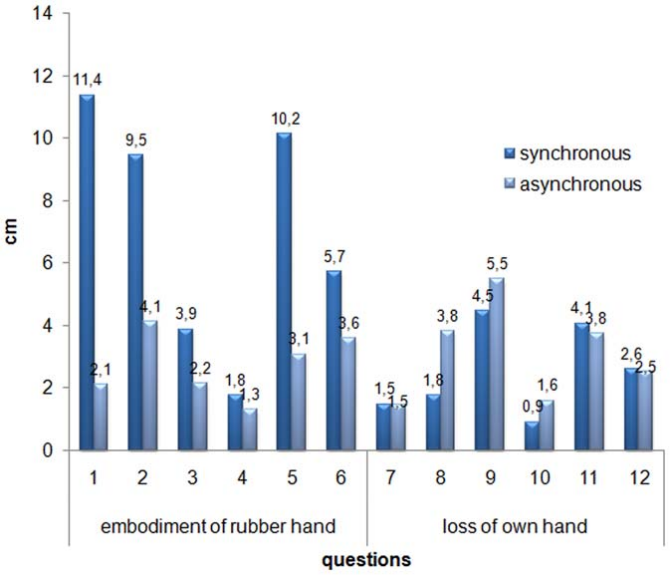
Questionnaire results. Mean level of agreement with the questionnaire statements (for specific questions, see Appendix S1).

The mean values of agreement for each question were analysed to characterise the subjective description of the RHI associated feelings. The results are graphically illustrated in Figure 4. An ANOVA was performed considering the variables *synchrony* of the training (synchronous/asynchronous) and *embodiment* (embodiment of the rubber hand/loss of own hand), according to the results of the principal components analysis by Longo and colleagues [16]. Both *synchrony* [F(1,14) = 21.42 p = .0004] and *embodiment* [F(1,14) = 7.42 p = .02], as well as their interaction were significant [F(1,14) = 21.93 p = .0003], revealing that the kind of visuo-tactile training influenced differentially the participants' agreement feelings. Overall, participants were more likely to agree with the questionnaire's statements after synchronous (5.18 cm) than asynchronous training (3.07 cm). The agreement was also stronger with statements related to embodiment of the rubber hand (5.04 cm) than those related to loss of their own hand (3.3 cm). The interaction critically revealed that after synchronous training participants agreed more strongly with questions related to embodiment of the rubber hand (7.1) with respect to questions related to loss of their own hand (3.27, p = .0002). The two components did not differ after asynchronous training, where the agreement was globally weaker (2.99 vs. 3.14, respectively).

In a separate analysis we focused on the two sub-components distinguished by Longo et al, [16] within the “embodiment of the rubber hand” general component, namely *location* of touch (questions 1–3) and *ownership* (questions 4–6, see Appendix S1), to assess whether they were equally involved during the induction of the RHI. To this aim, we performed an ANOVA exclusively on questions related to embodiment of the rubber hand with *synchrony* (synchronous and asynchronous) and *component* (location and ownership) as within-subject variables. As in the previous analysis the variable *synchrony* was highly significant [F(1,14) = 44.76, p = .00001], with stronger agreement after synchronous (7.10) than asynchronous training (2.99). Moreover, the significant interaction [F(1,14) = 6.02, p = .03] showed a more important contribution of the component *location* (8.27) than o*wnership* (5.94) in determining the higher mean in the synchronous condition (p = .006, by Newman-Keuls post-hoc test).

#### Proprioceptive judgment task

**Figure 5:**
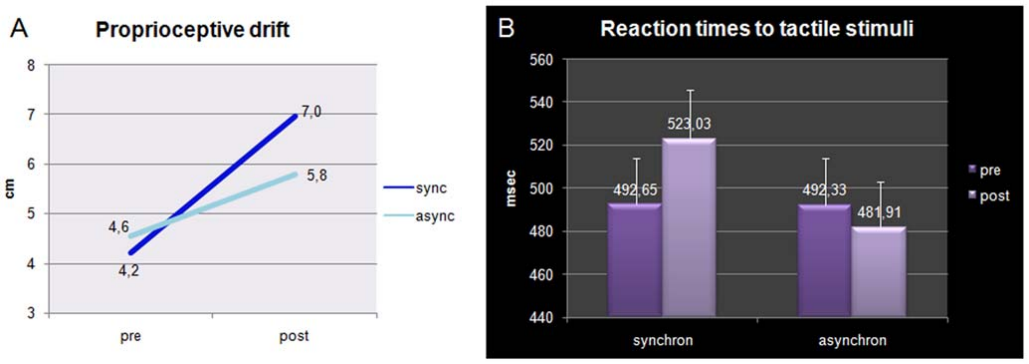
Somatosensory results experiment 1. Mean performances (in cm) in the proprioceptive judgment task (a) as a function of the session and synchrony of the visuo-tactile training; Mean RT performances (and S.E., in ms) in tactile task (b) as a function of synchrony of the visuo-tactile training and session.

The mean estimated location of the middle finger position of each participant's unseen hand was submitted to a repeated measures analysis of variance (ANOVA) with the variables *visuo-tactile training* (synchronous, asynchronous) and *session* (pre-, post-training). Results are shown in Figure 5a. The interaction between the two variables was significant [F (1,14) = 8.92, p = .01]. Participants erred toward the rubber hand and this drift increased both after synchronous (from 4.2 cm to 7.0 cm, p = .0002) and asynchronous training (from 4.6 cm to 5.8 cm, p = .004). Critically, however, this increase was significantly larger after synchronous than asynchronous training, as shown by a significant difference between errors after the two types of visuo-tactile training (7.0 cm vs. 5.8 cm, p = .006 by Newman-Keuls post-hoc test).

#### Tactile task

A similar ANOVA with the same variables was performed on the mean RTs to electro-cutaneous stimuli. Also on this variable the interaction *visuo-tactile training* and *session* was significant [F (1, 14) = 4.79, p = .046]. In particular, as illustrated in Figure 5b, RTs were slowed down with respect to the initial baseline after synchronous (from 492.6 to 523 ms, p = .04), but not after asynchronous training (from 492.3 to 481.9 ms, p = 0.4 by Newman-Keuls post-hoc test). Participants made very few detection errors (i.e., omissions) and the pattern of accuracy was not significantly different (varying from 89% to 84% in the synchronous condition, and from 91% to 90% in the asynchronous condition; interaction p = 0.3), thus excluding any speed-accuracy trade-off.

### Experiment 2

#### Proprioceptive judgment task

**Figure 6:**
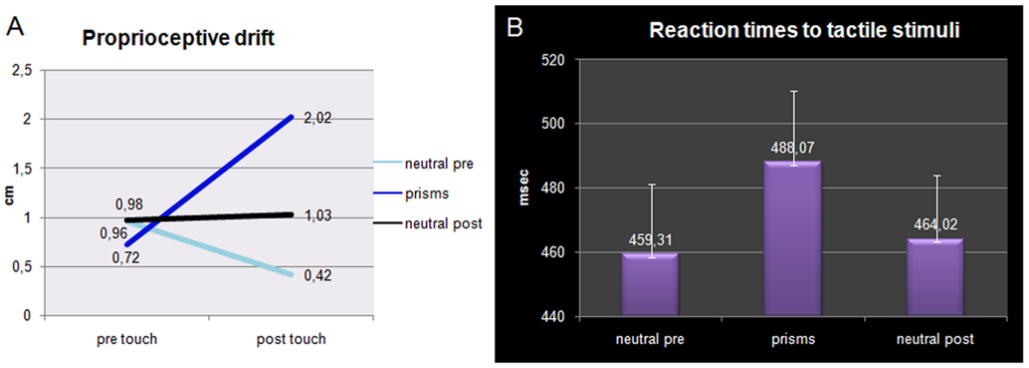
Somatosensory results experiment 2. Mean performances (in cm) in the proprioceptive judgment task (a) as a function of the visual conditions; Mean RT performances (and S.E., in ms) in tactile task (b) as a function of the visual conditions.

The mean values of the proprioceptive judgments made before and after every block were submitted to an ANOVA with *visuo-proprioceptive alignment* (neutral pre-prisms  =  match, prisms  =  mismatch, neutral post-prisms  =  match) and *session* (pre-, post-training) as variables. No main effect was significant, but the significant interaction [F (2,18) = 6.29, p = .008] revealed that the mean judgment of hand position was significantly more biased towards the optically displaced position of the participant's hand (i.e., leftwards) after the prismatic block (2.0 cm), than before the same block (0.7 cm, p = .02 by Newman-Keuls post-hoc test). No differences were present between judgments performed before and after wearing neutral goggles (see Figure 6a).

#### Tactile task

Mean RTs to electro-cutaneous stimuli were submitted to an ANOVA with *visuo-proprioceptive alignment* as within-subject variable (see Figure 6b). The *visuo-proprioceptive alignment* variable was significant [F (2.18) = 7.01, p = .006], showing that RTs were slower when participants wore prismatic goggles, that is in the condition in which vision and proprioception were misaligned, with respect to when they wore neutral goggles, where matching inputs were provided by vision and proprioception (neutral pre-prisms, mean = 459.3 ms, neutral post-prisms, mean = 464 ms, prisms, mean = 488 ms; p<.007 and .009 respectively for both comparisons between neutral goggles and prisms by Newman-Keuls post-hoc test). Again, the pattern of accuracy was not significantly different across conditions (88% in both neutral goggles conditions and 88% in prism condition), thus excluding any speed-accuracy trade-off.

#### Prismatic after-effect

As expected, a paired t-test between mean open-loop pointing performances at the beginning (mean = 0.13 cm) and at the end of the session (mean = 0. 48 cm) did not show any difference (t = −1.05, p = 0.31).

## Discussion

The question of how the human brain can generate a sense of ownership towards external body-parts, like rubber hands [2], [3], other people's faces [23], or even whole bodies [24], [25] has propelled the RHI [1] as the main tool for investigating bodily self-consciousness, and brought about new theories concerning whether it is conditional for self-consciousness to emerge to be spatially situated in the body [13], [26], [27]. A seminal approach in this respect is to determine the conditions and constraints for a fake body part (e.g. [28], [7] or entire body [29], [30] to be felt as one's own.

Another much less investigated perspective consists in establishing whether appropriating an alien body part would impact the sense of ownership of the corresponding real body part. Two interesting studies [16], [17] have recently addressed the question of disownership by assessing possible changes on the real hand either at the physiological and perceptual levels or at the introspective level. Thermal variations and altered temporal judgments were reported after RHI [17] and loss of one's hand is indeed part of the RHI experience [17]. One may then conclude that feeling ownership towards the rubber hand leads to implicit ‘rejecting’ one's real hand (but see [31]), which in turn induces somatosensory variations in one's real hand. However, here we provided evidence for an alternative explanation.

Experiment 1 revealed a strong RHI as proved by the subjective agreement with the questionnaire's statements following synchronous training and, more objectively, by the larger proprioceptive drift following the same training. This is in line with classical findings [1], [2], [32], [33] although it was induced here under peripheral vision of the rubber hand unlike most previous works (but see [5]). More importantly, the induction of the RHI altered tactile perception on the participant's real hand, as assessed by tactile RTs. This is the first measure of the RHI that is purely related to the critical, synchronous condition. There is indeed a proprioceptive drift both after synchronous and asynchronous stimulations, as compared with pre-test. The RHI is then typically measured on the basis of the relative difference between the two drifts. By contrast in the tactile task there was no effect of asynchronous stimulation on tactile perception, as compared with pre-test. It was only after synchronous stimulation that participants were slower in responding to supra-threshold touches delivered to their right, experimental hand. Hence, compared to the proprioceptive drift the slowing of tactile RTs seems to be highly selective for the phenomenon of the RHI. These results confirm our prediction that the RHI should affect tactile perception on the participant's real hand.

As shown by Longo and colleagues [16], several components are in play during the RHI experience. Hence the question arises as to which aspect induced by the illusion is responsible for the effect on somatosensation. If one focuses on the disownership component alone it would be tempting to ascribe the slowing of RTs to a form of artificially induced disownership of one's own hand, brought about by the illusion (see [17]). Once the hand no longer ‘belongs’ to the subject, tactile inputs arising from it may take longer to get access to conscious awareness. However, in the analysis of our questionnaire there was no indication of feelings of disownership for the real hand evoked by synchronous stimulation (see Figure 4). Instead, our findings revealed that a robust sub-component of the questionnaire is the spatial component of visual capture, i.e., the systematic alteration of the perceived *location* of touch. This finding has been documented consistently across studies, leading to the suggestion that the RHI includes a multisensory spatial mislocalization component due to visuo-proprioceptive conflict [34]. Once the illusion is established, touch is captured somewhere else by vision of the rubber hand. We therefore hypothesized that the slowing down of tactile processing found in Experiment 1 could originate from the spatial mismatch between visual and proprioceptive inputs related to the participant's hand location, rather than from hand disownership.

Experiment 2 aimed at more directly test the alternative hypothesis. Can visual-proprioceptive mismatch bring about the same results on speeded tactile detection? In Experiment 2 we induced a mismatch between visual and proprioceptive inputs about the position of the participant's real hand by using prismatic lenses diverting the visual location of the subject's hand 15° leftward to its proprioceptively defined location. As anticipated in the Introduction, prismatic lenses selectively affect visual spatial location of the hand without affecting the sense of ownership of the seen hand, which in this case remains unquestionable. If our alternative hypothesis were true, we predicted that RTs to tactile stimuli should be slowed while looking to one's own hand via the prisms.

This simple form of spatial conflict between visual and proprioceptive inputs was indeed sufficient to affect participants' tactile performance in a similar way to Experiment 1. RTs to tactile stimuli were longer during the conflicting than the non-conflicting conditions. Although direct comparison of performances across different groups should be interpreted with caution, we compared mean RTs across experiments. An additional t-test was performed between the mean differences due to the induction of the RHI (i.e., synch post - synch pre in experiment 1) and those due to prism exposure (i.e., prism condition - neutral condition in experiment 2). No significant difference was found (p = 0.9; means 30.38 and 28.76, respectively). In other words, the size of the effect was comparable when absolute group differences were subtracted.

It is worth noticing that we found slower RTs despite the fact that the subject's hand, as a result of the displacement of the fixation point, was seen in more central vision in the prismatic than in the neutral conditions. Our tactile task, not spatial in nature, was unlikely to cause visual enhancement of touch perception [35]–[37]. However, the visual position of the hand closer to fixation might have, if anything, cued tactile detection. Instead, negative effects were found on touch perception by maintaining unchanged the distance from fixation to the proprioceptive position of the hand (see [38], [22]).

In addition, we found a significant drift of the perceived position of the participant's hand towards its optically displaced visual position. Finally, the observed effects were independent of any change in egocentric reference system, as assessed by the open-loop pointing task. As expected, the short period of prisms wearing, during which touch perception was assessed, did not bring about any after-effect.

Our results are twofold. First, we showed that the RHI induced a slowing of tactile RTs. Second, we showed that the same effect was induced by wearing prism lenses. In a nutshell, what the two experimental set-ups have in common is the visuo-proprioceptive conflict. On the basis of these findings, we suggest that it is the visuo-proprioceptive conflict *per se* that modifies tactile perception, and not disownership (see Figure 1).

The present study aimed at testing the hypothesis that embodiment of a fake limb, induced via the RHI, determines the disembodiment of the subject's real limb, both at sensory and at the experiential levels. In particular, we assessed whether any disembodiment would be reflected by the participants' answers to a questionnaire and by altered performances at a relatively low level of somatosensory processing. To this aim, in Experiment 1 we measured changes in speeded tactile detection tasks for supra-threshold touches delivered to the participant's hand in the RHI context. Like in any RHI study, we found that synchronous cross-modal training produced a significant shift in the felt position of the participants' hand that was larger than that observed after asynchronous stimulation. More interesting, we found evidence that tactile RTs were slower after the induction of the RHI. Participants' tactile performance was selectively affected after synchronous visuo-tactile training (i.e., visual brushing of the fake hand concurrent with tactile brushing of the unseen real hand). The absence of changes in the asynchronous visuo-tactile training condition ruled out possible interpretations in terms of fatigue or unspecific test-retest effects on tactile RTs. Our results differ from those recently reported by Longo and colleagues [39] who found improved tactile perception in a Grating Orientation Test with near sensory threshold intensities, due to synchronous stroking as compared to asynchronous or no stroking (but see [40]). However, it must be noticed that in their study the rubber hand was reflected in a mirror and appeared as projected in a position coincident with the participant's real hand (i.e., no visual-proprioceptive mismatch). This arrangement caused a visual enhancement of touch. By contrast, in our study, participants saw the rubber hand 15 cm left from the real hand. The mismatch of the visual from the tactile/proprioceptive location of the hand makes the enhancement effect of touch by vision unlikely. Rather, it has a detrimental effect on the performance. As suggested by an anonymous reviewer, the role possibly played by shifts in covert attention should also be taken into consideration in the study of the RHI and its implications for ownership.

Overall, the findings of Experiment 1 concerning touch and proprioception confirm and extend those reported by Moseley and colleagues [17]. Yet, they may not be readily interpreted as tactile consequences of hand disownership. In the questionnaire, we found a very weak agreement with statements describing the loss of one's own hand, compared to ownership of the rubber hand. There was no significant difference, nor a trend for differential modulation of the agreement with disownership as a function of the synchronous vs. asynchronous condition. This result questions the disownership interpretation of the tactile disruption (model 1 in Figure 1). In addition, it should be emphasized that tactile disruption emerged in our data only for RTs, and not for accuracy, whereas hand disownership should in principle affect the overall capability to consciously perceive touches, thus increasing the number of omissions of tactile stimuli[14], instead of merely slowing down the process of an otherwise preserved detection.

In Experiment 2 we assessed an alternative explanation for the slowing of tactile RTs observed in Experiment 1. Instead of considering the tactile effect as the ultimate consequence of the causal chains induced by the RHI set up, we considered whether it could instead be a direct consequence of visual capture of touch (model 2 in Figure 1). Because of synchronous stroking of the unseen real hand and the visually seen rubber hand, any tactile stimulus on the real hand is visually localized in a position that is not congruent with the one coded via proprioception, thus giving rise to a highly unnatural conflict between the visual and the proprioceptive maps of the hand location. To solve this conflict the brain may diminish the weight attributed to somatosensory inputs [41], which may result in slower reaction times. To test this hypothesis we induced an artificial misalignment of the visual and proprioceptive position of the participants' hand by using prismatic goggles. We asked the participants to report unseen tactile stimuli delivered on their own hand while looking at it with prismatic lenses which displaced the position of the hand leftward of its real location. Participants were thus put in a conflicting situation that resembled in many ways the one produced under conditions of the RHI: (i) they felt touch on the seen hand; (ii) the seen hand was not congruent with proprioceptive signals; (iii) the seen hand was their own. Like in the RHI, the results showed slower RTs to tactile stimuli during this visual/proprioceptive conflict, compared to a condition where participants wore neutral goggles. Again, in Experiment 2 like in Experiment 1, the subjects' accuracy was not affected by this manipulation.

We suggest that what the two experiments have in common is that the participant's brain no longer ‘knows’ where the real hand exactly is. The conflict is similarly solved via a visual capture of proprioception, as shown by the proprioceptive drift exhibited by participants. The shift in the proprioceptive judgment was selectively present when the conflict was established, i.e., when subjects were wearing the prisms as compared to the two conditions when they wore the neutral goggles. It is interesting to note here that there can be visuo-proprioceptive conflict only if both sources of information concern the same object, namely one's own hand. This is a prerequisite to any multisensory integration. In our case, this implies that a precondition for the tactile effect and proprioceptive displacement to occur is the misalignment of visual and proprioceptive maps in the presence of *ownership*. Ownership of the seen hand seems to constitute the *sine qua non* condition. In Experiment 1 we indeed found that only when the hand was felt as one's own, as shown by the proprioceptive drift and questionnaire, the RTs slowed down. In the control condition with asynchronous training, the visuo-proprioceptive mismatch was the same as in synchronous training (Experiment 1) and in the manipulation with prisms (Experiment 2). However, in that case subjects did not feel ownership of the rubber hand, and neither the proprioceptive drift nor the slowdown in tactile processing was observed. As for disownership, it remains to be shown that the RHI does induce it and that it may affect somatosensory perception. We found that participants did not report feeling alienated from their hand and we showed that the effects on tactile perception can be explained without appealing to a denial of ownership towards one's hand. Hence it is still an open question whether the rubber hand literally replaces the real hand, and how to induce disownership artificially.

To sum up, tactile perception is impaired because the participants have lost their hand. They have lost it, not because their hand does not belong to them anymore, but because they no longer know where their hand is.

## Supporting Information

Appendix S1 Questionnaire
(0.03 MB DOC)
