# Supplementary material for: Losing One's Hand: Visual-Proprioceptive Conflict Affects Touch Perception
Source: PLoS One. 2009 Sep 7;4(9):e6920. doi: 10.1371/journal.pone.0006920 (PMC2732904; doi:10.1371/journal.pone.0006920)
Supplement: Appendix S1 — Questionnaire (0.03 MB DOC) [file pone.0006920.s001.doc]

**Appendix S1**

Questionnaire

| 1 | It seemed as if the touch I was feeling were caused by the paintbrush touching the rubber hand |
| --- | --- |
| 2 | It seemed as if I were feeling the touch of the paintbrush in the location where I saw the rubber hand touched |
| 3 | It seemed as if I were feeling the touch of the paintbrush somewhere between my own hand and the rubber hand |
| 4 | It seemed as if I might have more than two hands |
| 5 | I felt as if the rubber hand were my hand |
| 6 | It seemed as if the rubber hand began to resemble to my own hand, in terms of dimension, shape, skin tone or some other visual feature |
| 7 | I felt as if my own hand were turning "rubbery" |
| 8 | I felt as if my right hand were feeling less the touch of the paintbrush |
| 9 | I didn't know exactly where my right hand was located |
| 10 | I felt as if I were no longer able to move my right hand |
| 11 | I felt as if my right hand had disappeared |
| 12 | I felt as if my right hand were no longer in my control |
